# Isolation and Characterization of Diesel-Degrading Bacteria from Hydrocarbon-Contaminated Sites, Flower Farms, and Soda Lakes

**DOI:** 10.1155/2022/5655767

**Published:** 2022-01-21

**Authors:** Gessesse Kebede Bekele, Solomon Abera Gebrie, Eshetu Mekonen, Tekle Tafese Fida, Adugna Abdi Woldesemayat, Ebrahim M. Abda, Mesfin Tafesse, Fasil Assefa

**Affiliations:** ^1^Department of Biotechnology, College of Biological and Chemical Engineering, Addis Ababa Science and Technology University, Addis Ababa, Ethiopia; ^2^Department of Biology, Dire Dawa University, College of Natural and Computational Sciences, Dire Dawa, Ethiopia; ^3^Microbial,Cellular and Molecular Biology Department, Addis Ababa University, Addis Ababa, Ethiopia

## Abstract

Hydrocarbon-derived pollutants are becoming one of the most concerning ecological issues. Thus, there is a need to investigate and develop innovative, low-cost, eco-friendly, and fast techniques to reduce and/or eliminate pollutants using biological agents. The study was conducted to isolate, characterize, and identify potential diesel-degrading bacteria. Samples were collected from flower farms, lakeshores, old aged garages, asphalt, and bitumen soils and spread on selective medium (Bushnell Haas mineral salt agar) containing diesel as the growth substrate. The isolates were characterized based on their growth patterns using optical density measurement, biochemical tests, and gravimetric analysis and identified using the Biolog database and 16S rRNA gene sequencing techniques. Subsequently, six diesel degraders were identified and belong to *Pseudomonas*, *Providencia*, *Roseomonas*, *Stenotrophomonas*, *Achromobacter*, and *Bacillus*. Among these, based on gravimetric analysis, the three potent isolates AAUW23, AAUG11, and AAUG36 achieved 84%, 83.4%, and 83% diesel degradation efficiency, respectively, in 15 days. Consequently, the partial 16S rRNA gene sequences revealed that the two most potent bacterial strains (AAUW23 and AAUG11) were *Pseudomonas aeruginosa*, while AAUG36 was *Bacillus subtilis*. This study demonstrated that bacterial species isolated from hydrocarbon-contaminated and/or uncontaminated environments could be optimized to be used as potential bioremediation agents for diesel removal.

## 1. Background

Hydrocarbons, such as polycyclic aromatic hydrocarbons (PAHs), benzene, kerosene, and diesel are important organic pollutants and inputs for different industries, vehicles, and household activities as a source of energy [1–8]. Among these, diesel is massively used for engine, fuel, and industrial applications. It is one of the products of petroleum compounds formed during fractional distillation of crude oil and is composed of a mixture of carbon chains between 9 and 25 carbon atoms that may include both aromatic and aliphatic hydrocarbon components [4, 6, 9]. These hydrocarbon components can be discharged into the environment (groundwater, soil, and air) from different sources (point and nonpoint), such as garages, gas station services, chemical and petrochemical industries, agricultural waste, automobile exhaust spillage of petroleum, run-off asphalt pavements, vehicular emission, and combustion of fossil fuel [10–12]. This phenomenon may happen intentionally or accidentally mainly from anthropogenic activities because of urbanization, industrialization, and civilization [3, 10, 11, 13–15] and, to some extent, by natural disasters [16]. As a result, hydrocarbon-derived pollutants are immuno-toxicant, mutagenic, and carcinogenic to humans and animals and affect natural ecosystem functioning in many ways [2–4, 6, 7, 14, 15, 17–21].

There are different methods of mitigating hydrocarbon pollutions. These include mechanical, chemical, and biological approaches. The first two aforementioned means of mitigation of pollutants need high operational costs and are prone to secondary pollution that necessitates integrated pollution management to reduce and/or remove the toxic pollutants from the environment [13, 22]. On the other hand, the biological method (bioremediation) is a promising technology that is prominent, eco-friendly, cost-effective, efficient, and easily applicable for the treatment of hydrocarbon-contaminated environments [3, 8, 11, 15, 16, 19, 23–26], but it necessitates a long time for complete degradation of pollutants [27]. This approach mainly relies on two main techniques, namely, bioaugmentation and biostimulation [22]. Bioaugmentation involves an introduction of selected hydrocarbon-degrading microbial strains or consortia to the polluted environment to boost the already existing potential microbial communities for the biodegradation process [11, 18, 22, 23, 28]. Biostimulation, however, engages the amendment of macro- and micronutrients, sustains physical parameters (pH, temperature, and aeration), and supplies surface-active substances (surfactants) in contaminated sites to optimize soil conditions and enhance biodegradation by increasing the growth rate of indigenous (naturally occurring) hydrocarbon-degrading microorganisms [4, 6, 11, 17, 18, 22, 29]. This technique can be applied in situ and ex situ [28] to enhance biodegradation by increasing the bioavailability of the pollutants and the growth rate of native (indigenous and autochthonous) hydrocarbon-degrading microorganisms [23]. Thus, potential microbes use hydrocarbon-derived pollutants as a source of carbon and energy [27, 30] and/or cometabolites, finally leading to the complete mineralization of contaminants to carbon dioxide, water, mineral salts, and biomass [2, 3, 11, 13, 19, 25, 31].

Many studies showed that diverse microorganisms or microbial communities, namely, bacteria, fungi, yeasts, protozoa, and algae, play a great role in the biodegradation of hydrocarbon pollutants, and among those, bacteria are the most dominant and active degraders [3, 14, 15, 19, 21, 31]. The hydrocarbon-degrading bacteria are ubiquitous [6], of which the most known genera are: *Achromobacter*, *Marinobacter*, *Actinobacter*, *Alcaligenes*, *Mycobacterium*, *Arthrobacter*, *Bacillus*, *Rhodococcus*, *Corynebacterium*, *Micrococcus*, *Flavobacter*, *Nocardia*, *Bravibacterium*, *Streptococcus*, *Bacillus*, *Stenotrophomonas*, *Methylobacterium*, *Enterobacter*, and *Pseudomonas* [4, 5, 26, 31–34]. Their effectiveness for biodegradation and detoxification of hydrocarbon pollutants is because of their diverse enzymatic activities including hydrolases, oxygenase, demethylase, dehalogenases, transferases, and oxidoreductases that can catalyze different degradation routes aerobically or anaerobically [3, 4, 11, 15, 24, 30, 33, 35], as well as their effective reproduction potential [16]. These enzymes, which are often encoded by genes located on chromosomal or plasmid DNA [30, 35], can act on a diverse range of hydrocarbons via aerobic or anaerobic routes [3, 4, 11, 15, 24, 33]. The fast and absolute degradation of hydrocarbons or other organic pollutants is brought via aerobic conditions [21, 27, 30, 35, 36].

Aerobic biodegradation mostly utilizes oxygenase enzymes (monooxygenases and dioxygenases) [12, 26] for an oxidative attack of alkyl side chains and the hydroxylation of aromatic rings (benzene, toluene, xylene, and naphthalene). However, the anaerobic degradation is catalyzed by anaerobic or facultative bacteria using different final electron acceptors, such as sulfate, nitrate, iron, manganese, and CO_2_ [26, 30, 37]. The first step in oxidative biodegradation pathways is the activation of the ring for cleavage (*meta* or *ortho* cleavage) by hydroxylation using oxygenase enzymes [12, 35]. Both short- and long-chain hydrocarbons are oxidized to the corresponding alcohol that is later converted into aldehyde by alcohol dehydrogenase, and the aldehyde oxidized into the acid by aldehyde dehydrogenase. Consequently, the resulting fatty acids go through the ß-oxidation system to acetate (even-chain alkanes) and propionate (odd-chain alkanes). The hydrocarbon products are then subsequently oxidized into the Krebs cycle intermediates and eventually mineralized to CO_2_ and water [12, 38]. Therefore, indigenous microorganisms can degrade hydrocarbon-derived pollutants through natural remediation or attenuation into less or nontoxic forms in the environment [3, 10–12, 16]. However, natural attenuation is often limited when there is a lack of proper nutrient availability, high ability of microbial communities, and necessary catabolic genes for complete hydrocarbon degradation [39]. In addition, individual bacteria can metabolize only a limited range of hydrocarbon substrates, such as alkanes, and others that are paraffinic and aromatic, but a bacterial consortium with broad enzymatic capacity is required to synergistically degrade complex mixtures of pollutants [12, 16, 31]. Studies also showed that for successful biodegradation, the number of hydrocarbon-degrading bacteria should be in the range of 10^4^ to 10^7^ CFU per gram of soil [13] and considerably higher in hydrocarbon-contaminated sites [36].

However, the functioning of bacterial community structure and complete degradation is influenced by different factors such as the inherent genetic characteristics of the microbial population (catabolic gene or types of enzymes), several microbes (size), microbial mitigation or interaction (single strain or consortia), microbial diversity (bacteria, algae, and fungi), microbial competition (synergistic or antagonistic), the nature and characteristics of hydrocarbon pollutants (chemical structure, concentration, bioavailability, and toxicity level), the physical environment (nutrients, temperature, water activity, pH, soil moisture, and types of electron acceptors for respiration), and so on [4, 6, 8, 11, 12, 15, 16, 18, 19, 23, 25, 26, 39–42].

Many different genera of bacteria have been isolated from soil and aquatic environments and demonstrated to degrade hydrocarbons. Furthermore, identifying indigenous bacteria with diesel-degrading ability is essential as such isolates acclimatize to the local environment successfully when applied to reclaim polluted sites. In Ethiopia, garages, gasoline stations, car workshops, agricultural wastes, and industrial effluents are the most important nonpoint and point sources of pollutants that contaminate soil and water and affect the functioning of the ecosystem. However, regarding the biodegradation of hydrocarbons, there is a dearth of information on bacterial isolates that are native to the Ethiopian environment. Thus, the objective of this study was to isolate, characterize, and identify the potential diesel-degrading bacteria from hydrocarbon-contaminated samples from different study sites. Hence, indigenous diesel-degrading bacteria were isolated and screened for their efficacy and further characterized by cultural (colony characteristics), growth pattern (OD), biochemical tests, and identified using BioLog and 16S rRNA gene sequencing.

## 2. Materials and Methods

### 2.1. Study Area

Soil samples were collected from sites such as old aged garages (from the Addis Ababa region, namely, Amanuel and Akaki), old aged asphalt (from the Addis Ababa region, Amanuel), bitumen spill area (from the Addis Ababa region, Woira Sefer), and the Gallica flower farm located in Menagesha (22 km West of Addis Ababa) that were potentially exposed to hydrocarbon contamination. Sites such as Chitu Soda lake (180 km from Addis Ababa and located in the Southern Rift Valley of Ethiopia) have no known potential exposure to hydrocarbon contaminants. The soil samples were designated as AAUA (soil samples from Akaki/Amanuel garage), AAUAs (soil sample from Amanuel old aged asphalt site), AAUG (soil sample from Gallica flower farms), AAUW (sample from Woira Sefer bitumen soils), and AAUC (soil samples from Chitu soda lake).

### 2.2. Sample Size and Sampling Methods

Approximately 10 g of humid soil samples were collected from the topsoil (5–10 cm) of each of the selected study sites (in triplicate) using the simple random spatial sampling method. The samples were transferred into sterile polyethylene bags, labeled, kept in an icebox, and transported to the Microbial Biotechnology Laboratory at Addis Ababa Science and Technology University and stored in a refrigerator (EVERmed LR270W, Motteggiana (MN), Italy) at 4°C until use.

### 2.3. Enrichment of Diesel-Degrading Bacteria

The isolation of the hydrocarbon-degrading bacteria was undertaken by using an enrichment medium with the modified method of [13, 16, 19, 37, 39]. The enrichment medium or modified basal salt medium (BSM) contained (g/l of distilled water): KH_2_PO_4_ (1.36), Na_2_HPO_4_ (1.39), KNO_3_ (1.25), MgSO_4_ (0.06), CaCl_2_ (0.02), (NH_4_)_2_SO_4_ (7.7), NH_4_Cl (1.5), NH_4_NO_3_ (0.85), K_2_HPO_4_ (0.53), and 100 mL of a trace mineral solution containing 0.01 g of ZnSO_4_·7H_2_O, MnCl_2_·4H_2_O, H_3_BO_4_, CoCl_2_·6H_2_O, Fe_2_SO_4_·2H_2_O, CuCl_2_·2H_2_O, and NaMoO_4_·2H_2_O. The triplicate soil samples of each site were manually homogenized and sieved using a sterile 2 mm mesh screen. Then, 1 g of each sample was weighed and mixed into 9 mL of saline solution (0.99% of NaCl), from which 1 mL of the supernatant was transferred into 50 mL of enrichment medium supplemented with 0.5% (v/v) of diesel. The diesel used in this experiment was obtained from a local oil filling station (Jemal Tulu Dimtu Total Oil and Gas Station, Ethiopia), and it was filter-sterilized using 0.45 *μ*m diameter of the membrane filter [3] in 100 mL capacity Erlenmeyer flasks. The flasks were incubated in an intelligent thermostatic shake cultivation cabinet (ZHP-Y2112L series, Yangzhou Sanfa Electronic Co. Ltd., Jiangsu, China) with 150 rpm at 30°C, for 6 days [10]. Then, 10% (v/v) of the enriched culture was subsequently transferred into the enrichment media three times for refreshment.

### 2.4. Isolation and Plate Counting of Diesel-Degrading Bacteria

Following enrichment, the microbial cultures were prepared using appropriate dilutions using sterile saline solution (0.99% NaCl; 10^−1^, 10^−3^, 10^−5^, and 10^−7^), and from which 0.1 mL suspension was spread on Bushnell Haas mineral salt (BHMS) agar medium containing 0.5% (v/v) diesel. The medium contained MgSO_4_.7H_2_O (0.2 g/L), CaCl_2_ (0.02 g/L), KH_2_PO_4_ (1 g/L), K_2_HPO_4_ (1 g/L), and NH_4_NO_3_ (1 g/L) and 2 drops from 60% of FeCl_3_ at pH 7.2. Two controls (negative) were used, that is, BHMS media with diesel but not enriched culture and BHMS media with enriched culture but not diesel supplements. The plates were incubated for 6 days at 30°C to observe and determine the colony-forming units (CFU g^−1^). Isolates with distinct colonies were purified by streak plating onto a nutrient agar medium (HiMEDIA, Bengaluru, India). Then, the isolates were designated as AAU (Addis Ababa University) with their identification sites (A = Akaki and Amanuel garage; C = Chitu lake; G = Gallica flower farm; As = Amanuel old aged asphalt, and W = Woira Sefer bitumen soil) and respective identification numbers and preserved in 25% v/v glycerol at −20°C (IGnIS CHEST FREEZER C0450W, Milano, Italy) for a month. The preserved pure cultures were then subcultured and used as inoculants for the diesel biodegradation assays.

### 2.5. Bacterial Morphology and Biochemical Tests

#### 2.5.1. Morphological Characteristics

The isolates' morphological characteristics were studied using Gram's staining protocols and microscopy results [43].

### 2.6. Biochemical Tests

#### 2.6.1. Catalase Test

Three drops of hydrogen peroxide (3%) were added to the overnight grown culture in the test tubes and the formation of vigorous bubbles indicated catalase activity [5, 7].

#### 2.6.2. Casein Hydrolysis

Isolates were grown overnight in nutrient broth and inoculated onto skim milk agar (HiMEDIA) and incubated at 30°C for 48 h [5, 7]. The formation of a clear zone around the isolates against the white background indicated the casein hydrolysis activity of the isolates.

#### 2.6.3. Urease Test

The pure bacterial isolates were inoculated into urea broth (Difco, BD, Wokingham, UK) and incubated at 30°C for 24–48 h [7]. The change of color from yellow to pink indicated that there was urease production.

#### 2.6.4. Starch Test

Isolates were grown overnight in nutrient broth and inoculated onto a starch agar medium (Alpha Chemika, Mumbai, India) and incubated at 30°C for 48 h [5, 7]. The plates were flooded with Gram iodine. The formation of a clear area around the isolate against the blue-black background indicated starch hydrolysis.

#### 2.6.5. The Biodegradation Capacity of Diesel-Degrading Bacteria

Overnight bacterial culture [31] with a total plate count of 10^8^ cells/ml [38] for each isolate was inoculated into 100 BHMS broth containing 1% and 3% (v/v) diesel as substrate in 250 mL flasks and kept in a shaker incubator at 150 rpm at 30°C [12] for 5, 10, and 15 days. The growth (turbidity) was measured using a UV/Vis spectrophotometer (Mecasys, Optizen POP Series, K LAB, Daejeon, Mecasys Co. Ltd., South Korea) at 600 nm (OD_600_) in triplicate against BHMS medium as blank.

#### 2.6.6. Estimation of Diesel Biodegradation Efficiency by Gravimetric Analysis

The isolates amounted to 10^8^ cells/mL and were inoculated into 100 mL BHMS supplemented with 5% diesel dispensed in 250 mL conical flasks on a rotary shaker (150 rpm) and incubated at 30°C for 10 and 15 days. The residual concentration of diesel was assessed using the method with slight modification [17, 19, 40]. Thus, 1% 1N HCl was added into the culture to stop the bacterial activity, and the residual was extracted from the whole content using petroleum ether and acetone (1:1 v/v ratio). Then the flask was placed on the shaker at 120 rpm for 20 min, and the oil-containing solvent was collected from the upper portion of the flask and poured into the preweighted Petri plate [8]. It was repeated three times to ensure complete extraction. The extracted component was allowed to evaporate in a hot air oven at 72°C. Then, the residual diesel was calculated as the percentage of degradation using the following formula [8, 15, 20]:

(1) $$\begin{array}{c} \text{percentage degradation }\left(\%\right)=\frac{\left(\text{initial concentration of the diesel}-\text{final concentration of diesel}\right)}{\text{initial concentration of diesel}}*100. \end{array}$$

#### 2.6.7. Identification of Diesel-Degrading Bacteria Using BioLog

Preliminary identification of the isolates was made using Omnilog/BioLog systems according to the manufacturer's specifications (BioLog Inc., Hayward, CA, USA) at Sebeta National Animal Health Diagnostic Center, Ethiopia. The 96 wells of the microplates were filled with carbon sources and other nutrients and the utilization of carbon sources and/or resistance to inhibitory chemicals was colorimetrically determined using tetrazolium redox dyes. The bacterial isolates were grown on Biolog Universal Growth (BUG) agar medium using the protocol “A” that used inoculation fluid A (IF A) and a default protocol to identify the vast majority of bacterial species and then suspended in a special “gelling” inoculating fluid 3 (IF) at 90–98% cell density. The cell suspension was then inoculated into the GEN III microplate (100 *μ*l per well) and incubated at 33°C for 16 h. After incubation, the phenotypic fingerprints of purple wells were compared to BioLog's extensive species library (database of microorganisms) using a microplate reader.

### 2.7. Genetic Characterization of the Isolates

#### 2.7.1. Genomic DNA Extraction and PCR Amplification Using Bacterial Specific 16S rRNA Primers

The freeze-thaw technique was used to extract the genomic DNA from each pure bacterial colony for use as a template to amplify a bacterial domain-specific ca.1,500 bp 16S rRNA gene. For this purpose, the colony was suspended in 50 *μ*l sterile water and lysed by boiling for 5 min. It was then centrifuged at 12,000 ×g for 10 min, from which 1 *μ*l of the supernatant of lysed cells was transferred into 20 *µ*l of the PCR master mix [44]. The master mix consisted of 16.2 *µ*l PCR-grade water, 2 *µ*l of 10x PCR buffer (Life Technologies, Carlsbad, CA, USA), 0.4 *µ*l of 10 mM DNTP mix (Life Technologies), 0.4 *µ*l of 20 mg/ml bovine serum albumin (BSA), 0.8 *µ*L of 25 mM MgCl_2_, 0.08 *µ*l of 50 *µ*M of each 8F (5′–AGAGTTTGATCCTGGCTCAG–3′) and 1492R (5′–GGTTACCTTGTTACGACTT–3′), and dream Taq polymerase (Life Technologies). DNA amplification was performed using a thermocycler (Verti cycler, Applied Biosystems, Thermo Fisher Scientific, Waltham, MA, USA). The PCR cycling program was as follows: initial denaturation at 96°C for 10 min, 35 cycles of 95°C for 30 s, annealing at 56°C for 30 s, elongation at 72°C for 1 min, and a final extension of 7 min at 72°C. Reagent composition of the PCR reaction mixture (50 *μ*L) containing genomic DNA extract (5*μ*l), 10X Taq polymerase buffer, dNTPs mixture (2.5 pmol), each primer (20 pmol), and Taq DNA polymerase (2.5 U). Finally, the 16S rRNA PCR amplicons were purified using the Illustra Exostar DNA purification kit (GE Health Care) according to the manufacturer's specifications. The purified PCR product then underwent partial sequencing using the 8F primer (monodirectional sequencing) at the sequencing facility of Leibniz Institute DSMZ (German Collection of Microorganisms and Cell Cultures, Germany). The 16S rRNA amplification was performed using the bacterial universal oligonucleotide primers 8F and 1492R using the Verti cycler PCR system (Applied Biosystems) as described by [45].

#### 2.7.2. Nucleotide Sequencing and Phylogenetic Analysis

Partial 16S rRNA gene sequencing was performed using the Illumina/Solexa sequencing facility, as described by [46] and the raw DNA chromatogram sequences were viewed and edited using Sequence Alignment Editor Version 7.0.5.3 [47] and stored in FASTA format. The sequences were compiled and compared to the NCBI (http://www.ncbi.nlm.nih.gov) DNA sequence database using BLASTn to verify proximate phylogenetic positions.

### 2.8. Data Analysis

Numerical data were analyzed by analysis of variance (ANOVA) followed by a multiple comparison test (Duncan) with SAS statistics software (version 9.1.3; 2003, Cary, NC, USA), considering statistically significant differences to be those with a *p*-value <0.0001 of potent diesel-degrading bacteria. The phylogenetic tree was constructed in Molecular Evolutionary Genetics Analysis X (MEGA X; Pennsylvania State University, USA) with bootstrap values of 1,000 replications using the maximum likelihood method [48] and Kimura-2 parameter model [32].

## 3. Results

### 3.1. Isolation of Diesel-Degrading Bacteria

Nineteen diesel-degrading bacteria were isolated from the enrichment culture of different sampling sites (Table 1). The data showed that diesel-degrading bacteria were recovered from old aged asphalt sites, garage sites, and bitumen soil, which was expected to have exposure to hydrocarbon contamination, and from Gallica flower farm, which uses different agrochemicals containing polycyclic hydrocarbons. In addition, diesel-degrading bacteria were also recovered from Chitu soda lake that has minimal or no known history of previous direct hydrocarbon contamination.

### 3.2. Bacterial Identification and Characterization

#### 3.2.1. Characterization of Isolates Based on Cell Morphology and Biochemical Tests

In this study, 19 bacterial isolates were characterized using Gram's staining and biochemical tests (Table 1). Based on Gram's reaction, the majority of the bacteria (85%) were Gram-negative and rod-shaped, whereas 15% were Gram-positive rods. The isolates were also characterized based on standard biochemical tests, and all isolates were catalase positive, except AAUG10 (*Roseomonas cervicalis*). In addition, the majority of diesel degraders (68%) were capable of casein hydrolysis, excluding *Providencia rettgeri*, *Achromobacter xylosoxidans*, and *Stenotrophomonas maltophila*. The data also showed that 42% of the isolates were urease-positive and three Gram-positive *Bacillus* spp. were able to hydrolyze starch.

#### 3.2.2. Identification of Diesel-Degrading Species Using BioLog

The GEN III MicroPlate test panel provides a standardized micromethod to profile and identify a broad range of Gram-negative and Gram-positive bacteria based on 65.5 to 99.9% accuracy of identification of the species within genera (Table 1). Thus, the identified bacterial genera were *Pseudomonas* spp., *Roseomonas* spp*., Bacillus* spp*., Providencia* spp., *Achromobacter* spp., and *Stenotrophomonas* spp.

#### 3.2.3. Diversity of Diesel-Degrading Bacteria

Among the isolated species, *Pseudomonas aeruginosa* and *Stenotrophomonas maltophilia* accounted for 42% and 16%, respectively (Table 2). Besides, *Bacillus cereus* and *Providencia rettigeri* each accounted for the third group (11% of the distribution). The isolates were recovered from different sites that were predominately contaminated with hydrocarbon components or had no history of direct contamination of hydrocarbon constituents.

### 3.3. Screening of Isolates for Effective Diesel Degradation

#### 3.3.1. Diesel Biodegradation (1%)

**Figure 1:**
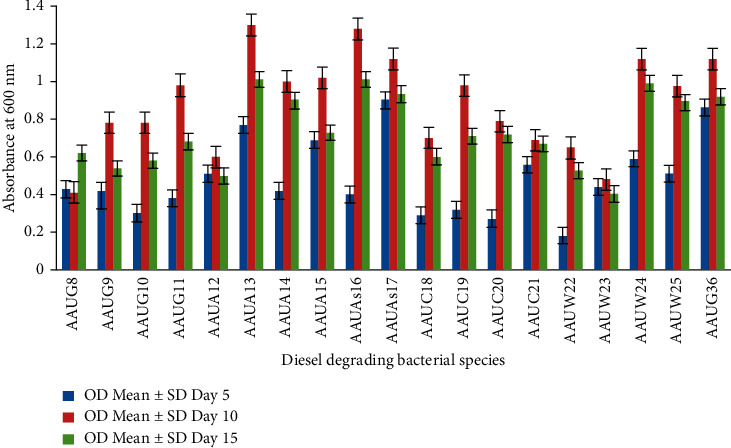
Growth capacity of isolates on diesel (1% concentration at different growth periods).

Bacteria utilize diesel for their growth, energy, and an increase in biomass [1]. The growth or increase in biomass is indicated by turbidity in the growth medium (BHMS). In this study, the growth pattern (effective degradation) of 19 bacterial isolates was enumerated on a BHMS medium supplemented with 1% diesel. The isolates showed significant growth on the 10^th^ day of incubation ranging from an OD of 0.41 ± 0.002 to 1.3 ± 0.004, indicating significant differences (*p* < 0.0001) in their ability to degrade diesel. Among the isolates, *B. cereus* (AAUA13) showed an OD value of 1.3 ± 0.004, followed by *A. xylosoxidans* (AAUAs16) with an OD value of 1.28 ± 0.002. Their degrading potential reached a peak on the 10^th^ day of growth incubation, which was threefold higher than the 5^th^ day of incubation (Figure 1). Similarly, *P. aeruginosa* (AAUW24), *B. subtilis* (AAUG36), *P. rettgeri* (AAUAs17), *P. aeruginosa* (AAUG11, AAUC19, and AAUW25), and *S. maltophilia* (AAUA14 and AAUA15) showed no significant difference in their growth. In this study, it was investigated that only one isolate, *P. rettgeri* (AAUG8), showed an increased growth measured in terms of OD from the 10^th^ day of incubation (0.41 ± 0.002) to the 15^th^ day of incubation (0.62 ± 0.002).

#### 3.3.2. Diesel Biodegradation (3%)

**Figure 2:**
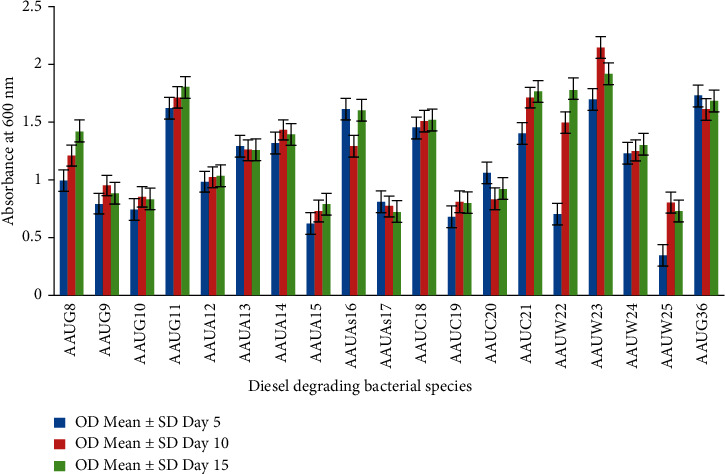
Growth capacity of isolates on diesel (3% concentration at different growth periods).

The growth pattern of bacterial isolates on hydrocarbon degradation on the diesel medium (3%) was also studied (Figure 2). The maximum growth was recorded on the 10^th^ day of incubation. Isolate AAUW23 (*P. aeruginosa*) showed a significant growth capability with an OD value of 2.14 ± 0.016 (*p* < 0.0001) compared to other isolates. An earlier study also showed that *P. aeruginosa* is efficient for the degradation of high concentrations of hydrocarbon contaminants [12]. In addition, the remaining isolates identified as *P. aeruginosa* (AAUC21, AAUG11, and AAUW22), *P. viridilivida* (AAUC18), and *S. maltophilia* (AAUA14) also showed modest diesel biodegradation activities with OD values ranging from 1.43 ± 0.003 to 1.71 ± 0.022. In a previous study, *S. maltophilia* was identified as a key hydrocarbon-degrading bacterium [2]. Some isolates also showed increased activity as the incubation period continued above 10 days, with a significant difference of OD value. Accordingly, *S. maltophilia* (AAUC20), *Providencia rettgeri* (AAUG8), *A. xylosoxidans* (AAUAs16), and *P. aeruginosa* (AAUW22) were identified as potential diesel degraders with OD values of 0.92 ± 0.075; 1.42 ± 0.047; 1.6 ± 0.022, and 1.78 ± 0.038, respectively. Therefore, *P. aeruginosa* considerably showed effective diesel degradation capacity. This could be because *P. aeruginosa* has a unique adaptive potential to survive in a range of diverse conditions including environments that harbor substantial concentrations of hydrocarbon sources such as diesel [19].

### 3.4. Gravimetric Analysis for Diesel Biodegradation Efficacy

**Figure 3:**
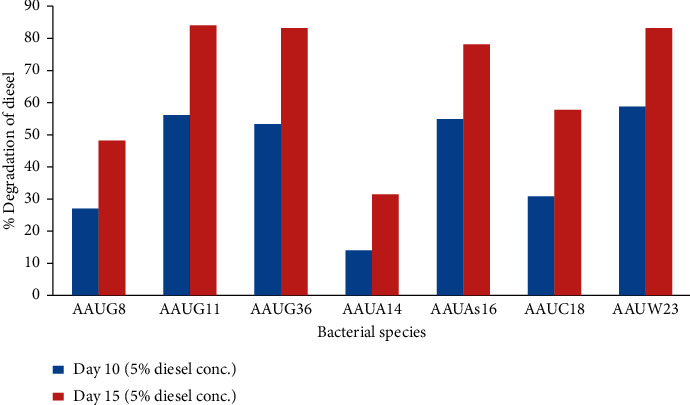
Gravimetric analysis for diesel degradation.

Seven bacteria isolates were found to grow and showed effective degradation competence in BHMS media containing 3% diesel concentration. These selected potential isolates were then provided with 5% diesel as a growth substrate and gravimetric analysis was performed on the 10^th^ and 15^th^ day of incubation (Figure 3). The results showed that two isolates of *Pseudomonas* spp. (AAUW23 and AAUG11) and *B. subtilis* (AAUG36) showed 83.6%, 84%, and 83% diesel degradation efficacy, respectively, on the 15^th^ day of incubation. The remaining isolates *P. viridilivida* (AAUC18), *P. rettigeri* (AAUG8), and *S. maltophila* (AAUA14) showed lower degradation efficiency for diesel (58%, 48%, and 31.6%, respectively) for the same day of incubation. The previous study by [17] also showed that the maximum degradation of diesel observed after 15 days of incubation was 53% when grown at 0.5% diesel concentration. Therefore, in this study, diesel was degraded at a high concentration (5%) and short exposure time.

### 3.5. 16S rRNA Sequences and Phylogenetic Analysis of Selected Isolates

Three of the most efficient bacterial isolates (designated as AAUW23, AAUG36, and AAUG11) that showed maximum diesel-degrading capability were selected upon the gravimetric analysis method, and their 16S rRNA was sequenced. The partial 16S rRNA sequences of the three bacterial isolates were submitted to the NCBI, and their accession numbers were obtained as MT669825 for AAUW23, MT669830 for AAUG36, and MT669831 for AAUG11. The 16S rRNA sequencing and phylogenetic data analysis of these three isolates using BLAST searches confirmed that the isolates are closely related to some of the 16S rRNA sequences of the cultured bacterial taxon in the Genbank database. Consequently, two isolates, AAUW23 and AAUG11, belonged to gamma subdivisions of *Proteobacteria*, while AAUG36 belonged to *Firmicutes* (Table 3).

**Figure 4:**
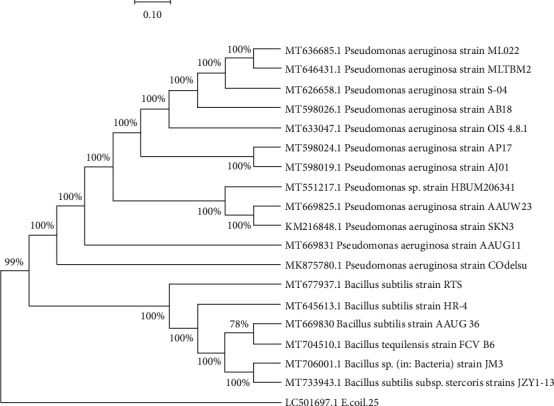
Phylogenetic tree based on partial bacterial sequences of the 16S rRNA region for the two *Pseudomonas isolates* and one *Bacillus subtilis* (bold and coded with the initials “AAU”) and accession numbers of the 16S rRNA are followed by species names. Numbers at nodes indicated bootstrap values for each node out of 1,000 bootstrap resembling. The phylogenetic tree was constructed in MEGA X using the maximum likelihood method [48] and Kimura-2 parameter model [32]. The *Escherichia coli* partial sequence was used as an out-group.

The phylogenetic tree was constructed in MEGA X using the maximum likelihood method, and it depicted that bacterial isolates AAUW23 and AAUG11 could well cluster with *P. aeruginosa*, while the isolate AAUG36 could relate with *Bacillus* spp. (Figure 4).

The constructed phylogenetic tree depicted that AAUG11 (MT669831) and AAUW23 (MT669825) shared 98% nucleotide identity and ≥99% similarity with other existing bacterial 16S rRNA sequences retrieved from the database. The isolate AAUG36 (MT669831) isolated from the Gallica flower farm soil sample was affiliated to *P. aeruginosa* strain SKN3 (MK216848.1) and strain COdelsu (MK875780.1) with a similarity of 99% previously isolated from plant phyllosphere and crude oil samples, respectively. The other isolate AAUW23 (MT669825) obtained from the bitumen soil sample also closely related to *Pseudomonas* spp. strain SKN3 (MK216848.1) and strain HBUM206341 (MT551217.1) previously described from environmental samples. In addition, the isolate AAUG36 (MT669830) was isolated from the Gallica flower farm and formed a common lineage with the strain *Bacillus tequilensis* FCV B6 (MT704510.1) with 99.34% similarity (bootstrap value of 78%) previously isolated from disinfectant-contaminated biofilm sample. In this study, therefore, *Pseudomonas* and *Bacillus* are the prevailing diesel-degrading bacterial genera detected in hydrocarbon-contaminated areas such as bitumen soil and flower farms. The present study revealed that bacteria inhabiting various hydrocarbon-contaminated soils/sediments could rapidly degrade diesel.

## 4. Discussion

Microorganisms play a vital role in the biodegradation (bioremediation) of hydrocarbon pollutants in a polluted milieu [49]. In this study, the bacterial isolates were recovered from soil samples of known or unknown hydrocarbon-contaminated environments using BHMS medium supplemented with 0.5% of diesel as a carbon source to enrich their growth pattern and determine their degrading potential. The population density enumerated from all sampling sites is within the recommended number of 10^4^ to 10^7^ CFU per gram of soil for successful hydrocarbon biodegradation [11]. However, there were differences in the number of bacterial isolates in the sampling sites. The fact that different numbers of colonies were obtained from these sites might be associated with the diversity of bacteria capable of degrading hydrocarbons and their derivatives [19]. In addition, a greater number of diesel-degrading bacteria could be recovered from garage sample sites and other various petroleum compound contaminated sites [3–5, 12, 20, 31]. This could be associated with the potential of bacterial survival on different types of hydrocarbon components such as aliphatic (diesel) and aromatic (monocyclic or polycyclic) hydrocarbons [3]. However, indigenous microorganisms that can degrade these pollutants through natural attenuation are very low [11, 16]. In addition, the current work demonstrated that hydrocarbon-degrading bacteria are also isolated from nonhydrocarbon-contaminated sites, such as soda lake (Chitu). In agreement with this, studies also showed that several strains of hydrocarbon-degrading bacteria have also been isolated from an environment with no known hydrocarbon contamination [34, 50]. This could be due to the existence of hydrocarbons from natural and anthropological origin or produced by the degradation and synthesis processes of some microorganisms [51, 52], and such natural environments are expected to contain highly reduced forms of hydrocarbon that are important to support microbial communities as good sources of carbon and energy [14, 29, 36, 51].

The majority of isolated diesel-degrading bacteria were Gram-negative, mainly belonging to five genera, namely, *Pseudomonas* spp., *Stenotrophomonas* spp., *Providencia* spp., *Roseomonas* spp., *and Achromobacter* spp., and found to be 47, 16, 11, 5, and 5% of the total isolates, respectively. Other studies also showed that the Gram-negative species of the genus *Pseudomonas* spp. (38.94%) and *Achromobacter* spp. (7.96%) were characterized as diesel degraders [20]. In addition, among the total isolates identified in this study as diesel degraders, 16% were found to be Gram-positive isolates, which belong to species of the genus *Bacillus*. The diesel-degrading bacterial species were also characterized using some standard biochemical tests based on their catalytic activities. Some isolates showed positive results for the degradation of hydrogen peroxide, casein, starch, and urea, but others did not. This could be a preliminary indication that the isolates have diverse enzymes for catalyzing the degradation of various and/or specific substrates. They were also identified using BioLog data of which the majority of isolates were represented by *Pseudomonas* spp. with 84.9 to 98.1% accuracy. The next dominant diesel-degrading species was *S. maltophilia* (78.7–97.5%), followed by *Bacillus* spp. (65.5–87.5%), *P. rettigeri* (85.6–99.9%), *R. cervicalis* (70%), and *A. xylosoxidans* (95.7%). *P. aeruginosa*, *B. cereus*, *S. maltophilia*, *A. xylosoxidans*, and *P. rettgeri* were recovered from garages, old aged asphalt, and bitumen soil environments that are contaminated with hydrocarbon components by anthropological activities. Other related studies also confirmed that *P. aeruginosa*, *S. maltophilia*, and *B. cereus/subtilis* were also isolated from a wide variety of aliphatic and aromatic hydrocarbon-contaminated soils [12, 19]. In addition, *P. rettgeri*, *P. aeruginosa*, and *R. cervicalis* were isolated from flower farms, while *P. aeruginosa*, *P. viridilivida*, *and S. maltophilia* were also recovered from the Chitu soda lake site, which has no direct contact with hydrocarbon components. From the current and other previous studies, it could be recognized that *P. aeruginosa* is potentially obtained from various soil environments, mainly due to its ubiquity in terms of its diverse metabolic capability for diesel degradation.

The growth capacity of the isolates was then detected at different diesel concentrations (1, 3, and 5%). At 1% diesel concentration, two bacterial isolates, *B. cereus* (AAUA13) and *P. aeruginosa* (AAUAs16), showed significant growth patterns on the 10th day of incubation. This indicates that *Bacillus* spp. and *Pseudomonas* spp. displayed superb diesel degradation potential [19, 42]. In addition, *P. rettgeri and S. maltophilia* were identified as potential diesel degraders [1, 24]. Notably, the current study demonstrated that species of the genus *Pseudomonas*, *Achromobacter*, *Providencia*, and *Stenotrophomonas* were identified as potential candidates for diesel degradation/utilization compared to the other isolates for longer culture time (15 days) and a higher concentration of the substrate (3% diesel). In addition, the study also showed that the isolates performed better activity on the degradation of 3% diesel concentration than 1% concentration on the same day of incubation. This is for the reason that an increase in OD with an increase in diesel concentration in the growth medium indicates an increase in hydrocarbon-degrading bacterial population as they use it as a carbon and energy source [14]. *P. aeruginosa* (AAUW23 and AAUG11) were the most efficient biodegraders of diesel. Many studies also confirmed that *Pseudomonas sp*. shows superb diesel degradation efficacy [1, 12, 14, 15, 19–21, 40]. This is because it is an oleophilic microorganism [16] and has metabolic versatility, or it may be symbiotically associated in soil [8], or it produces a biosurfactant, which effectively makes diesel more available for utilization [4, 9, 18, 40]. In addition, other studies also showed that such bacterial species possess enzyme systems to degrade and utilize diesel oil as a source of carbon and energy [14, 19]. In addition, *B. subtilis* (AAUG36) was also identified as a potent bacterial species for the degradation of diesel. The study also showed that this species can be found in different environments due to its ability to produce endospores to pass harsh environments [13, 22] and surface-active substances (biosurfactants) to decrease surface and intersurface tension and increase the bioavailability of contaminants for efficient biodegradation [22]. This biological characteristic is important to augment the bioavailability of poorly accessible diesel and to enhance the biodegradation rate. Therefore, this study indicated that *P. aeruginosa* and *B. subtilis* showed maximum degradation with a higher concentration of diesel (5%) and without using any synthetic surfactants.

The 16S rRNA gene sequence alignments using BLASTn search in NCBI as well as the BioLog identification system for the three species of potential diesel-degrading bacterial isolates (AAUW23, AAUG36, and AAUG11) revealed that the isolates were found to exhibit 99% and above identity for existing cultured bacteria in the database. The 16s RNA gene partial sequencing **(**Table 3**)** identified two different bacterial genera, namely, *Pseudomonas* spp. and *Bacillus* spp., and both isolates, AAUW23 and AAUG11, were recognized as *P. aeruginosa* and isolate AAUG36 as *B. subtilis.* Interestingly, the sequences of these isolates were aligned with the analogous sequences of several other known hydrocarbon-degrading organisms, and the resulting phylogenetic tree indicated that these isolates were grouped into the phyla *Proteobacteria* (AAUW23 and AAUG11) and *Firmicutes* (AAUG36), with Gammaproteobacteria being the most represented class (Figure 4). The report from earlier studies also described that the phylum *Proteobacteria*, in most cases, has characteristics that are closely associated with aliphatic and aromatic hydrocarbon-degrading organisms [12, 20].

## 5. Conclusion

Bioremediation is one of the current approaches in environmental microbiology or environmental biotechnology that has been exercised for the reduction and/or removal of hydrocarbon pollutants. Microorganisms, typically bacteria that have particular metabolic capacities, are essential for the biodegradation of hydrocarbon pollutants. The present study provides a scientific investigation on diesel-degrading bacteria obtained from different soil environments based on culture-dependent techniques. It was found that potential bacteria that could degrade diesel would readily be isolated from hydrocarbon-contaminated soil samples or other natural environments that have no direct contamination with hydrocarbon residuals. This could be a groundwork indication for a possible search of potential bacterial isolates for the bioremediation of hydrocarbon-contaminated environments. The 16S rRNA gene sequencing and phylogenetic tree construction inferred that the potential bacterial isolates AAUW23 (MT669825) and AAUG11 (MT669831), closely affiliated to species of the genus *Pseudomonas*, and AAUG36 (MT669830), which is affiliated to *Bacillus*, might be able to predominately survive and thrive in 1, 3, and 5% (v/v) diesel. The isolates also exhibited maximum diesel degradation efficiency using the gravimetric analysis method. Therefore, this study attests that bacterial species inhabiting different habitats are considered potential biological agents for the efficient biodegradation of diesel. This study also adds to the existing body of knowledge contributing to further improvements in the study towards minimizing environmental pollution contaminated with hydrocarbon components such as diesel.

## Figures and Tables

**Table 1:** Morphological and physiological characteristics of diesel-degrading bacteria isolated from different sampling sites.

Isolates code	BioLog ID	Site	Gram's	Shape	Catalase	Urase	Casein	Starch
AAUG8	*Providencia rettgeri*	Flower	−	Bacilli	+	+	−	−
AAUG9	*Pseudomonas aeruginosa*	Flower	−	Bacilli	+	−	+	−
AAUG10	*Roseomonas cervicalis*	Flower	−	Coccobacilli	−	+	+	−
AAUG11	*P. aeruginosa*	Flower	−	Bacilli	+	−	+	-
AAUA12	*Bacillus cereus*	Garages	+	Bacilli	+	+	+	+
AAUA13	*B. cereus*	Garages	+	Bacilli	+	+	+	+
AAUA14	*Stenotrophomonas maltophila*	Garages	−	Bacilli	+	+	−	−
AAUA15	*S. maltophila*	Garages	−	Bacilli	+	+	−	−
AAUAs16	*Achromobacter xylosoxidans*	Asphalt	−	Bacilli	+	−	−	−
AAUAs17	*P. rettgeri*	Asphalt	−	Bacilli	+	−	−	−
AAUC18	*Pseudomonas viridilivida*	Soda lake	−	Bacilli	+	+	+	-
AAUC19	*P. aeruginosa*	Soda lake	−	Bacilli	+	−	+	−
AAUC20	*S. maltophila*	Soda lake	−	Bacilli	+	−	−	−
AAUC21	*P. aeruginosa*	Soda lake	−	Bacilli	+	−	+	−
AAUW22	*P. aeruginosa*	Bitumen	−	Bacilli	+	−	+	−
AAUW23	*P. aeruginosa*	Bitumen	−	Bacilli	+	−	+	−
AAUW24	*P. aeruginosa*	Bitumen	−	Bacilli	+	−	+	−
AAUW25	*P. aeruginosa*	Bitumen	−	Bacilli	+	−	+	−
AAUG36	*Bacillus subtilis*	Flower	+	Bacilli	+	−	+	+

**Table 2:** Diversity and community structure of isolates from hydrocarbon-contaminated sites and nonpolluted natural sites (Chitu Soda lake).

Genus of the isolates	Distribution (%)	Species of the isolates	Distribution (%)	Species distribution (%)	
				Contaminated Sites	Noncontaminated Sites
*Pseudomonas*	47	*P. aeruginosa*	42	31.5	10.5
		*P. viridilivida*	5	0	5
*Bacillus*	16	*B. cereus*	11	11	0
		*B. subtilis*	5	5	0
*Providencia*	11	*P. rettgeri*	11	11	0
*Roseomonas*	5	*R. cervicalis*	5	5	0
*Stenotrophomonas*	16	*Stenotrophomonas maltophilia*	16	11	5
*Achromobacter*	5	*A. xylosoxidans*	5	5	0

**Table 3:** Phylogenetic affiliation of 16S rRNA partial sequences of three bacterial isolates.

Isolate code	Accession number	Top-hit Taxon	GenBank accession	Identity (%)	Taxonomy
AAUG11	MT669831	*P. aeruginosa*	MT646431.1 MT636685.1 MT598024.1 MT626658.1 MT598019.1	99.69%, 99.69%, 99.69% 99.69% 99.69%	Bacteria; Proteobacteria; Gammaproteobacteria; Pseudomonadales; Pseudomonadaceae; *Pseudomonas*
AAUG36	MT669830	*B. subtilis*	MT645308.1 MT704510.1 MT706001.1 MT733943.1 MT677937.1	99.43%, 99.43%, 99.43% 99.43% 99.43%	Bacteria; Firmicutes; Bacilli; Bacillales; Bacillaceae; *Bacillus*
AAUW23	MT669825	*P. aeruginosa*	MT598024.1 KM216848.1 MK875780.1 MT626658.1 MT633047.1	99.23% 99.34% 93.34%, 99.23% 99.23%	Bacteria; Proteobacteria; Gammaproteobacteria; Pseudomonadales; Pseudomonadaceae; *Pseudomonas*

## Data Availability

The data that support the findings of this study are available on request from the corresponding author. However, the partial 16S rRNA sequences of bacterial isolates have been deposited in the National Center for Biotechnology Information (https://www.ncbi.nlm.nih.gov/) under the accession numbers MT669825, MT669830, and MT669831.
